# Context is key: normalization as a novel approach to sport specific preprocessing of KPI’s for match analysis in soccer

**DOI:** 10.1038/s41598-022-05089-y

**Published:** 2022-01-21

**Authors:** Ashwin A. Phatak, Saumya Mehta, Franz-Georg Wieland, Mikael Jamil, Mark Connor, Manuel Bassek, Daniel Memmert

**Affiliations:** 1 Institute of Exercise Training and Sport Informatics, , German Sports University, Cologne, Germany; 2 Institute of Physics, University of Freiburg, Freiburg, Germany; 3 School of Health and Sports Sciences, University of Suffolk, Ipswich, UK; 4 Natural Computing Research and Applications Group, Smurfit School of Business, University College Dublin, Dublin, Ireland

**Keywords:** Computational science, Statistics

## Abstract

Key Performance Indicators (KPIs) have been investigated, validated and applied in multitude of sports for recruiting, coaching, opponent, self-analysis etc. Although a wide variety of in game performance indicators have been used as KPIs, they lack sports specific context. With the introduction of artificial intelligence and machine learning (AI/ML) in sports, the need for building intrinsic context into the independent variables is even greater as AI/ML models seem to perform better in terms of predictability but lack interpretability. The study proposes domain specific feature preprocessing method (normalization) that can be utilized across a wide range of sports and demonstrates its value through a specific data transformation by using team possession as a normalizing factor while analyzing defensive performance in soccer. The study performed two linear regressions and three gradient boosting machine models to demonstrate the value of normalization while predicting defensive performance. The results demonstrate that the direction of correlation of the relevant variables changes post normalization while predicting defensive performance of teams for the whole season. Both raw and normalized KPIs showing significant correlation with defensive performance (p < 0.001). The addition of the normalized variables contributes towards higher information gain, improved performance and increased interpretability of the models.

## Introduction

‘Context is king’ is a term used in popular media. The term suggests that trying to analyze or understand any information without placing it in the right context can lead to potentially false conclusions. There has been a rising emphasis on the use of ‘Big Data’ in sports. Although there is huge potential for knowledge discovery in specific sports, it is still unclear how this vast amount of data can be used efficiently^1^. Sport specific context and knowledge becomes crucial for interpreting this ‘big data’. Notational data and match statistics have been used across sports by professional teams for analyzing self and opponents, developing tactics, recruiting and to obtain an overall competitive edge^2^. Sports researchers are also using this data to increase the knowledge of physical, technical and tactical aspects of the game. A wide variety of performance indicators have been used in the sports industry and in research for analyzing different aspects of sports^3,4^. Some amount of this data is now readily available as opensource data from companies such as FBref (https://fbref.com/) and Whoscored (https://www.whoscored.com/). Some of the indicators such as, shots are also present in contextualized form such as percentage of shots on target. Nonetheless, there are many notational statistics which are used without accounting for phase of play and specific context of the play^5,6^.

Events and actions in most team sports seem to have non-linear relationships^7^. Machine Learning and Artificial Intelligence and Machine Learning (AI/ML) methods used in data science offer a wide set of tools to model these non-linear relationships^8^. Due to the ‘black-box’ nature of these AI/ML tools, it becomes imperative that the input features (performance indicators) incorporate sports specific context within themselves. Currently, there is limited research that considers this context while analyzing Key Performance Indicators (KPIs). Pre-processing, although being an important step in data science has so far only been sparingly implemented in notational analysis in invasion sports. Pre-processing in terms of feature engineering has shown to change or improve performance of AI/ML models both in terms of interpretability and predictive power^9^. A study, which analyzes out of possession fouls as a performance indicator in the top five leagues in European football, shows that the direction of correlation with end of season performance is inverted form negative to positive when accounted for possession percentage of the respective teams. The same. In addition, the study outlines the importance of normalizing relevant KPI’s with respect to the phase of play and further elaborates possible extensions for incorporating sport specific knowledge while analyzing KPI’s in soccer^10^.

Normalization of performance indices is a standard procedure in sport science to assure comparability between subjects. In physical performance analyses, parameters like oxygen consumption (VO2)^11^ or maximal strength^12^ are usually divided by the athlete’s body mass. Another example of normalization is the use of percentage of maximal values of such parameters to account for individual capacities when characterizing training load^13^. Thus, absolute values of performance indicators on the individual level gain in value when context information is added. It seems fair to assume that the same is true for performance indicators on a team level. As previously mentioned, accounting for ball possession as a proxy for teams changes the interpretation of fouls as a KPI ^10^. However, little work has been done on the effect of normalization on different KPIs.

Multiple studies have been conducted for analysis of position data, devising player ratings, data visualization and data driven performance analysis but, so far none of them consider the requirement of preprocessing the features while analyzing performance indices^14–17^. Using such raw data can lead to evening out of the results and lead to issues such as reliability, validity and precision of findings ^6,18^. There seems to be a need for a developing a method, which provides an outline for effectively analyzing notational data with built in domain specific knowledge. The main aim of the present study is to outline the importance of normalizing KPI’s according to sport specific context for soccer and to provide for the first-time data preprocessing technique for effectively incorporating sport specific context within the sport. The study also demonstrates how applying additional context to raw data through normalization, using previously vetted preprocessing techniques can ultimately result in a more informative analysis, thereby aiding the development of players, team tactics and recruitment procedures.

## Methods

### Sample

End of the season performance statistics from the English Premier League, Italian Serie A, French League one, Spanish LaLiga and the German Bundesliga were obtained for the seasons from 2017–18 to 2020–21 (up until February) from the open-source website FBref.com (https://fbref.com/). Permission for the use of open-source data is available under the creative commons license as specified by FBref (https://www.sports-reference.com/data_use.html). All data preprocessing and statistical modelling was performed using Python 3.7 (H2O library) and R 4.0.

### Normalization

Defensive performance statistics for blocking and tackles + interceptions along with average possession data was used for each team, where blocking and tackles + interceptions are described in accordance to the definitions on StatsBomb (https://github.com/statsbomb/open-data/blob/master/doc/StatsBomb%20Open%20Data%20Specification%20v1.1.pdf). It is important to note that all defensive statistics recorded happen while the team in question is in the defensive phase i.e., without the possession of the ball. The time spend in the defensive phase is different for every team, hence, normalizing the above indices to account for out of possession time becomes crucial^10^. The general formula for all normalized defensive KPIs is as follows:

1 $$Norm\,Defensive\;KPI =Defensive\;KPI /(1-Possession\;Percentage /100)$$

In the current study formula (1) is used for two specific cases of Blocks and Tackles + Interceptions to demonstrate the value of normalization as a generalized process while analyzing all out of possession defensive actions.

2 $$Norm\,Blocks =Blocks /(1-Possession\;Percentage /100)$$

3 $$Norm\,Tackles\;and\;Inerceptions =( Tackles+Inerceptions)/(1-Possession\;Percentage /100)$$

Thus, four performance indicators, i.e., Blocks, Tackles + Interceptions, Norm Blocks, and Norm Tackles + Interceptions were obtained. In all the above equations the ‘Possession Percentage’ refers to average end of season possession percentage which the team in question accumulated, ‘Blocks’ refers to the number of shots blocked by the team at the end of the season and ‘Tackles + Interceptions' refers to the addition of tackles and interceptions performed by the concerned team at end of each season.

### Statistical modelling

Linear regressions were performed with expected goals against (xGA) as the dependent variable in each of the models. The independent variables in the first two models were Blocking performance (Blocks and NormBlocks) and Tackling performance (Tackles + Interceptions and NormTackles + Inerceptions).

In contrast to linear regression, a gradient boosting machine algorithm is capable of modelling nonlinear relationships and can account for multicollinearity issues within the independent variables (IV’s)^19^. Hence 4 gradient boosting regressions were performed to predict xGA. The first model included just the Raw KPIs, the second included the normalized KPIs and the third used both raw and normalized KPIs as input features. A fourth GBM model was built with the raw KPIs with the addition of the team’s possession as the third input variable. In order to examine the combined effect of all KPI’s and rank their importance, H2O AutoML was used for finding the best GBM model with R^2^ (explanation of variance) as the optimization variable. fivefold cross validation (CV) was performed on all models for comparing out of sample validity of each model.

### Ethics approval

Not applicable as the study was done on open-source, validated data without participants.

### Consent for publication

Obtained under creative commons license.

## Results

Table 1 below shows the results of linear regression predicting xGA as a function of the blocking performance of the team in question. The model significantly predicts xGA (*p* < 0.01 and R^2^ for cross validation = 51.1% ± 12.1%). Both Blocks (*p* ≤ 0.001) and NormBlocks (*p* ≤ 0.001) seem to significantly predict xGA. An increase in one standard deviation of total Blocks shows an increase of 10.97 xGA. While one standard deviation increase in NormBlocks shows a decrease of 4.01 xGA.

**Table 1:** xGA vs blocking performance (R^2^ = 51.1 ± 12.1%).

Names	Coefficients	Standard error	Z value	P value	Standardized coefficients
Intercept	7.392	2.226	3.320	< 0.01–04	44.754
Blocks	0.109	0.006	19.237	< 0.001	10.975
NormBlocks	−0.019	0.003	−7.025	< 0.001	−4.008

Table 2 below shows the results of linear regression predicting xGA as a function of the tackling and interception performance of the team in question. The model significantly predicts xGA (*p* ≤ 0.001 and *R*^*2*^ for cross validation = 39.7% ± 12.1%). Both Tackles + Interceptions (*p* ≤ 0.001) and Norm Tackles + Interceptions (*p* ≤ 0.001) seem to significantly predict xGA on average. An increase in one standard deviation of total Tackles + Interceptions shows an increase of 10.09 xGA. While one standard deviation increases in NormTackles + Interceptions shows a decrease of 5.52 xGA on average.

**Table 2:** xGA vs tackling performance (R^2^ = 39.7 ± 12.1%).

Names	Coefficients	Standard error	Z value	P value	Standardized coefficients
Intercept	11.265	2.825	3.987	< 0.01	44.753
Tkl + Int	0.072	0.004	16.653	< 0.001	10.092
NormTkl + Int	−0.016	0.002	−9.115	< 0.001	−5.524

Table 3 below shows the results of GBM model, which predicts xGA by using only raw KPIs (Blocks and Tackles + Interceptions) as the IV’s. 58.77% of the variance predictive power of the model is predicted by raw blocks and 41.2% by Tackles + Interceptions.

**Table 3:** GBM results for xGA vs raw KPIs.

Variable	Relative importance	Scaled importance	Percentage (%)
Blocks	95,117.367	1.000	58.8
Tkl + Int	66,722.398	0.702	41.2

Table 4 below shows the results of GBM model, which predicts xGA by using only KPIs (NormBlocks and NormTackles + Interceptions) as the IV’s. 65.8% of the variance predictive power of the model is predicted by raw blocks and 34.2% by Tackles + Interceptions.

**Table 4:** GBM results for xGA vs normalized KPIs.

Variable	Relative importance	Scaled importance	Percentage (%)
NormBlocks	72,164.851	1	65.8
NormTkl + Int	37,500.191	0.520	34.2

Table 5 below shows the results of the gradient boosting regressor, which predicts xGA by combining the IVs in the above two models (Blocks and NormBlocks), Tackling performance ((Tackles + Interceptions) and (NormTackles + Inerceptions)). The model suggests that the highest percentage contribution of the KPI’s while explaining the variance in the model comes from Blocks (54.3%) and lowest form NormTackle + Interception (9.4%). Overall, the raw raw KPI’s contribute higher than the normalized ones but all selected KPI’s seem to contribute to more than 9.4% of the variance in the model.

**Table 5:** GBM results for xGA vs combined KPIs.

Variable	Relative importance	Scaled importance	Percentage (%)
Blocks	90,860.570	1.000	54.4
Tkl + Int	36,498.390	0.402	21.8
NormBlocks	24,005.883	0.264	14.4
NormTkl + Int	15,775.142	0.174	9.4

Table 6 below shows the results of the gradient boosting regressor, which predicts xGA by adding Possession and an additional IV to the raw KPIs (Blocks and Tkl + Int). The model suggests that the lowest contribution while explaining the variance in xGA comes from the team’s possession (15.1%) followed by Tackles + Interceptions (18.6%), while the highest contribution is from the blocks (66.2%).

**Table 6:** GBM results for xGA vs KPIs and possession.

Variable	Relative importance	Scaled importance	Percentage (%)
Blocks	108,474.078	1.000	66.25
Tkl + Int	30,484.479	0.281	18.61
Possession	24,762.047	0.228	15.12

Table 7 shows the cross-validation results of all the 6 models. The combined GBM model with normalized and raw KPIs shows the highest prediction accuracy and reliability (R^2^-CV = 57.83 ± 2.37) as compared to the raw KPI model (R^2^-CV = 49.90 ± 7.35), the normalized model (R^2^-CV = 37.16 ± 4.11) and the model which accounts for possession as an additional feature (R^2^-CV = 56.96 ± 2.57).

**Table 7:** Cross validation results for all models.

Model	Train (R^2^%)	Test (CV (R^2^% ± SD%))
Blocking performance LR	55.30	51.50 ± 12.07
Tackling performance LR	42.84	39.70 ± 12.16
Raw KPIS GBM	61.76	49.90 ± 7.25
Normalized KPIs GBM	45.53	37.16 ± 4.11
Combined performance GBM	70.11	57.83 ± 2.37
KPIs and possession GBM	66.72	56.96 ± 2.57

## Discussion

The aim of the study was to outline the importance of incorporating domain specific knowledge while analyzing KPI’s in soccer using preprocessing (Feature Engineering). The study outlined one such normalization technique to obtain, out of possession defensive KPI’s (NormDefensiveKPIs) based on previous findings^10^. It was observed that the model built using a GBM algorithm consisting of both raw and normalized KPIs outperformed all other models. This suggests that normalization of defensive KPIs using the outlined techniques adds interpretability in terms of football specific features without compromising the predictive performance of the model (see Tables 5 and 7). Feature Engineering to incorporate domain specific knowledge into input features is a common practice in applied machine learning but has only been used in sports analytics to a limited extent ^20,21^. Current study through possession-based normalization emphasizes the potential and the need for developing domain specific features in sports to effectively exploit the full potential of machine learning techniques.

The results of the linear regressions performed for blocking performance suggested that both Blocks and NormBlocks significantly predicted defensive performance, but the direction and magnitude of the correlations was flipped when the blocks were normalized. Number of ‘end of season blocks’ for a team seemed to be positively correlated with the xGA of that team (hence negatively with defensive performance) while NormBlocks were negatively correlated with xGA (positively with performance). This confirms recent work, that shows a negative correlation between defense KPIs and performance (i.e., chance of promotion) when analyzing absolute values^22^. However, when adding the context information of team strength, it becomes clear, that this is but half of the truth. As better teams take part in more offensive actions, like shots, worse teams get more chances for defensive actions, like blocks. But being able to block shots, regardless of team strength, seems to be an appropriate defense strategy as it prevents the attacking team from scoring. This is valuable information for coaches, as previous research suggests that situations where blocks occur (i.e., shot from opponent) need to be prevented.

The raw KPI’s available on open-source websites are counts of actions. Although, new performance indicators such xGA, percentage of shots on goal, save percentage and more do exist there are still a multitude of defensive KPI’s which can be improved by using gameplay information such as phase of play (in possession or out of possession) to normalize these individual KPI’s. Raw KPIs can be misleading as a team that performs more tackles, blocks and interceptions may be interpreted as the worse team, but once possession of the team is accounted for, the team which performs more successful defensive actions seems to be performing better (see Tables 1 and 2). Performance in soccer can be considered a complex dynamical system, whereby multiple interacting variables can potentially constrain performance at any given time. It is safe to assume that a multitude of performance statistics may show multi-collinearity with each other and the relationships may be both linear and nonlinear in nature ^18^.

Machine Learning (ML) algorithms such as Gradient Boosting Machine (used in current study), Random Forest, Support Vector Machines, Neural Networks etc. are capable of handling multicollinear features effectively, which may not be the case with generalized linear models ^19^. ML algorithms are also capable of generating non-linear models, which can potentially outperform generalized linear models in certain cases and may be closer to the true data generation process ^23^. ML algorithms combined with k-fold cross validations can successfully be used for assessing the out of sample validity of the model, thus providing an alternative to p-values and significance testing ^24^. One issue with using ML models is lack of interpretability. Generally, ML models provide high predictive performance and low transparencey which can be a challenge, but they do provide variable importance for the used models, which can be used to rank the effect of each feature while predicting the performance ^25,26^.

The generalized normalization process in the current study provides a partial solution to this problem in the specific case of defensive soccer KPI’s. The study suggests that defensive KPI’s be normalized with respect to possession as a preprocessing step to generate KPI’s which build in the time in/out of possession into the variables themselves. These variables can be initially analyzed using an interpretable model to obtain the direction of relationship with the selected performance measure and then Normalized plus the Raw KPI’s be fed into a combined ML model for improving its performance. By obtaining variable importance from the ML model with the prior knowledge regarding the KPI’s a more interpretable and higher performing model can be built. The nature of normalized KPI’s is such that they provide information as how the team performed without the ball, which is relevant while analyzing defensive KPI’s in particular. Such a normalization procedure also adds to recent work on understanding collective team behavior in football by adding the role-specific context to a performance model that would otherwise not be as representative of the task as it would include non-defensive phases of play in defensive KPI’s ^27^. In an attempt to allow research to be easily applicable to practice, this approach can help provide more domain-specific information, such as phase-of-play based distinctions, in order to further develop training designs that are most representative of the actual task thereby streamlining statistical research, training as well as competitive performance ^28^.

Furthermore, extensions of the normalization procedure to non-trivial cases are possible. This study used defense KPI’s on a season level and normalized them with overall season ball possession, which can be a limitation. More granular approaches could assess KPIs on a match or even player level. On a match level, normalization variables such as ranking, points in season or betting odds^29^ can potentially be used. On individual level, normalization might enable the analysis of both offensive and defensive performance of players for talent identification and scouting. For example, the individual KPI of tackles won, which is the proportion of successful tackles over the total amount of tackles, could be normalized by the time out of possession to gain a more detailed understanding of individual KPI’s. This analysis could be further enhanced if other data sources, such as positional data could be used. Then, even normalization over the number of possible tackles the player could have made would be possible. The same is true for offensive player-specific KPI’s, such as take-on or pass percentage. Individual pass statistics could e.g., be normalized again by the team’s possession share in order to gain an understanding of the true passing quality of the player.

## Conclusion

The findings of the current study suggest the requirement of building in sport specific knowledge on raw game statistics while analyzing and interpreting them. There can be further domain specific transformations that could be done on an individual player and even individual play level to make the results of traditional and ML models more predictable. This may provide further insight into the gameplay processes and bridge the theory practice gap. Such models can potentially be used in recruiting, tactics formations, team selection, coaching, scouting etc.

The current study uses aggregated data for three seasons across top five European soccer leagues, on a team and season level which may be susceptible to misinterpretation due to Simpson’s paradox^30^ another limitation may be the data gathering process although data from companies such as StatsBomb (current study) and Opta (StatsPerform) has been validated there still can be possibilities for error due to the manual data collection techniques and lack of transparency while building models such as player ratings and expected goals. The inclusion of normalization of variables seems to be effective in increasing model performance and reliability but further detailed studies need to be performed on a match level across separate leagues and with more KPI’s to reinforce the results of the current study. Furthermore, analogous non-trivial preprocessing techniques need to be explored which allow direct interpretation of the variables themselves sports specific language facilitating the application of AI/ML in the sports industry.

## Data Availability

Data was obtained from open-source websites under creative commons license.
